# A Simplified Frailty Measure Improves Risk Stratification in Patients Undergoing Percutaneous Mitral Valve Repair

**DOI:** 10.1002/jcsm.70138

**Published:** 2025-12-01

**Authors:** Clemens Metze, Elric Zweck, Christos Iliadis, Maria Isabel Körber, Matthieu Schäfer, Ralf Westenfeld, Amin Polzin, Marcel Halbach, Stephan Baldus, Roman Pfister

**Affiliations:** ^1^ Department of Cardiology, Heart Center Cologne University of Cologne, Faculty of Medicine and University Hospital Cologne Germany; ^2^ Division of Cardiology, Pulmonology and Vascular Medicine Heinrich Heine University, Medical Faculty Düsseldorf Germany

**Keywords:** frailty, MitraClip, percutaneous mitral valve repair, risk score

## Abstract

**Objective:**

The aim of this study was to examine frailty measures for risk prediction in patients undergoing percutaneous mitral valve repair (PMVR).

**Background:**

Performance of current risk prediction models based on organ morbidity is only moderate.

**Methods:**

The frailty domains exhaustion, slowness, inactivity, weakness, and unintentional weight loss were prospectively assessed in patients undergoing PMVR. The association with mortality and heart failure hospitalization (HFH) during a median follow‐up of 506 days was examined and relevant domains were assessed for additive predictive performance over the MitraScore.

**Results:**

Five hundred twenty‐four patients were included in the study (mean age [SD]: 77 (9) years, 54.6% male). The risk of mortality and mortality/HFH were significantly increased in patients with exhaustion (hazard ratio: 2.24 [95% CI: 1.63–3.08]/1.83 [1.21–2.77]), slowness (2.74 [1.75–4.30]/1.92 [1.34–2.75]), inactivity (1.96 [1.26–3.05]/1.61 [1.12–2.31]) and weakness (1.83 [1.12–2.96]/1.34 [0.92–1.95]), but not in patients with weight loss (1.24 [0.80–1.92]/1.12 [0.78–1.61]). A simplified frailty (S‐frailty) measure derived from the domains exhaustion and slowness was associated with mortality (4.45 [2.42–8.19]) and mortality/HFH (2.60 [1.64–4.13]) independently of clinical risk factors and the MitraScore, respectively. Prediction of mortality by the MitraScore was significantly improved when adding S‐frailty (Harrell's C 0.645 vs. 0.700, *p* = 0.002). Classification of 1‐year mortality risk by the MitraScore improved when adding S‐frailty (net reclassification index 0.21, *p* = 0.012 and integrated discrimination index 0.07, *p* < 0.001).

**Conclusions:**

A simplified frailty measure comprising self‐reported exhaustion and gait speed significantly improved risk prediction in patients undergoing PMVR. Assessment of frailty measures should be part of the decision‐making process and multiparametric risk scores in patients with severe mitral regurgitation.

## Abstract 2

We assessed the Fried frailty criteria in 524 patients undergoing percutaneous mitral valve repair and derived a simplified frailty measure comprising self‐reported exhaustion and gait speed, which was significantly associated with 1‐year mortality and mortality or heart failure hospitalization independently of clinical risk factors and the well‐established MitraScore. Additionally, the simplified frailty measure significantly improved risk prediction of the MitraScore. These findings provide first evidence supporting the assessment of frailty measures as part of the decision‐making process and multiparametric risk scores in patients with severe mitral regurgitation.

**Abbreviations**
COPD: Chronic obstructive pulmonary disease
HFH: Heart failure hospitalization
HR: Hazard ratio
LVEF: Left ventricular ejection fraction
MR: Mitral regurgitation
MVARC: Mitral Valve Academic Research Consortium
NT‐proBNP: N‐terminal pro‐brain natriuretic peptide
NYHA class: New York Heart Association class
PMVR: Percutaneous mitral valve repair
RAS: Renin‐angiotensin‐system
S‐frailty: Simplified frailty
TAVR: Transcatheter aortic valve replacement
TTE: Transthoracic echocardiography

## 1 Introduction

Percutaneous mitral valve repair (PMVR) has been shown to be an effective and safe therapeutic option in patients with symptomatic mitral valve regurgitation (MR) who are inoperable or at high surgical risk [1]. Given the high morbidity in patients selected for PMVR, an individualized risk–benefit estimation is essential prior to the treatment decision [2]. However, there is still a lack of means for accurate risk prediction in patients undergoing PMVR. Risk scores, such as the well‐established MitraScore, which have been validated in or derived from patients undergoing PMVR [3, 4, 5, 6] are focusing on cardiovascular and noncardiovascular organ morbidity. Accordingly, the MitraScore includes age, left ventricular ejection fraction (LVEF), anaemia, kidney function, peripheral arterial disease, chronic obstructive pulmonary disease (COPD), high dose of diuretics and no therapy with renin‐angiotensin‐system (RAS) inhibitors [6]. However, the risk scores ignore more holistic measures of patient health status that might be more relevant for outcomes in these patients.

Current guidelines recommend assessment of frailty by validated instruments during the clinical evaluation process of patients with valvular heart disease [7]. Frailty is a complex clinical syndrome determined by age, multimorbidity, nutrition and psychosocial factors, which collectively contribute to reduced physiological reserves and accumulation of functional deficits [8]. Albeit a generally accepted definition of frailty is lacking, physical frailty according to Fried is one of the most used frailty measures in studies and clinical routine. Physical frailty according to Fried is defined by the presence of distinct domains such as exhaustion, weakness, slowness, low activity levels and weight loss [9]. Frailty is a strong risk factor for mortality and rehospitalization in elderly people, patients with cardiovascular disease and patients undergoing cardiovascular interventions and cardiac surgery [8, 10, 11, 12]. However, the prognostic impact of frailty components is weakly characterized in patients undergoing PMVR so far.

We have shown earlier that frailty assessed by different measures is common in patients undergoing PMVR and associated with short‐ and mid‐term mortality [13, 14]. However, the prognostic contribution of individual frailty domains is unclear and might be of differential relevance in PMVR patients. In this context we have recently shown that inactivity, exhaustion and slowness are significantly improved shortly after PMVR therapy, which might attenuate their long‐term prognostic impact [15]. We hypothesize that only selected frailty components are of prognostic relevance and that these might add predictive value to existing risk scores.

The aim of this study was to examine the impact of individual frailty domains on outcomes in a large prospective cohort of patients undergoing PMVR and to evaluate whether selected frailty domains improve risk discrimination based on clinical routine parameters.

## 2 Methods

### 2.1 Patients

All consecutive patients who were admitted for nonurgent PMVR between May 2014 and December 2019 at the Heart Center of Cologne University Hospital were eligible for this prospective observational study. Exclusion criteria were an age of < 18 years and impossible or refused consent. All patients had symptomatic MR grade 3 or 4 with a treatment indication according to current guidelines [16] and were discussed in the interdisciplinary heart team consisting of cardiac surgeons, interventional and noninterventional cardiologists. After evaluating the surgical risk based on validated scores and clinical variables, the decision on interventional mitral valve repair was made. The interventions were performed with the MitraClip system (Abbott Vascular, Santa Clara, California, USA), the PASCAL system or the Cardioband Mitral Repair system (both Edwards Lifesciences Corp., Irvine, California, USA). The study was approved in its present form by the local ethics committee of the Medical Faculty of the University of Cologne (Reference 14‐116).

### 2.2 Baseline Assessments

Baseline clinical characteristics were collected before PMVR from the medical record. Euroscore II was calculated online (www.euroscore.org/calcold.html). Transthoracic echocardiography (TTE) with quantification of MR was performed before the procedure and at discharge according to guideline recommendations [16]. Laboratory findings were also obtained before the procedure. Frailty was assessed using the following five domains according to Fried [9]:

1. Self‐reported exhaustion: ‘In the past week (for 3 days or more) I felt everything I did was an effort, and I was not able to brace myself up for anything’
2. Slowness: Walking speed for 4.57 m > 6 s
3. Inactivity: Positive response of ‘strongly’ or ‘very strongly’ to the following question: ‘In the past month, did your heart failure prevent you from living the lifestyle you wanted, by requiring you to lie down or sit down to rest during the day?’
4. Weakness: Grip strength of the dominant hand measured by dynamometer averaged over 3 passes < 18 kg for women and < 30 kg for men
5. Unintentional weight loss: More than 5% of body weight or more than 4.5 kg within the past 12 months

Assessment of inactivity in our study was modified from the original Fried scale using a question from the Minnesota Living with Heart Failure questionnaire. This might be a reasonable approach because activity levels in heart failure patients show a high correlation with symptom burden‐based questionnaires [17]. Patients meeting at least three domains were classified as frail.

### 2.3 Follow‐Up

Prespecified complications were collected according to the Mitral Valve Academic Research Consortium (MVARC) [18], including mortality, neurological complications, heart failure‐associated rehospitalization and vascular complications requiring intervention. Long‐term follow‐up regarding mortality and heart failure‐related rehospitalization was collected by chart review and telephone call with patients, relatives or treating primary care physicians.

### 2.4 Statistics

Patient baseline parameters and outcomes were compared according to the presence or absence of the five frailty domains. When normally distributed, continuous variables were reported as mean and standard deviation. Statistical significance of differences was calculated using the Student *t* test. When not normally distributed, variables were presented as median and interquartile range (IQR) and differences across groups were tested using the Mann–Whitney *U* test. Because the distribution of NT‐proBNP levels was skewed, natural logarithmic (ln) transformation was performed. For nominal as well as ordinal data, the absolute number and percentage were given. The statistical significance of differences was calculated using the chi‐square test. For cells with an expected frequency less than five, Fisher's exact test was used. For factor level comparisons of survival distribution, we used the log‐rank test. Univariate and multivariate Cox regression analysis was used to examine the association between frailty domains and clinical outcomes. Multivariate models included all baseline characteristics reported in Table 1, which showed a significant association (*p* < 0.05) with mortality in univariate analysis.

**TABLE 1:** Baseline characteristics by status of individual frailty domains.

		Exhaustion			Slowness			Inactivity			Weakness			Weight loss		
		Positive *n* = 223	Negative *n* = 114	*p*	Positive *n* = 138	Negative *n* = 199	*p*	Positive *n* = 138	Negative *n* = 199	p value	Positive *n* = 205	Negative *n* = 132	*p*	Positive *n* = 143	Negative *n* = 194	*p*
Age, years		77.7 ± 8.5	78.4 ± 8.7	0.419	79.7 ± 6.9	76.7 ± 9.4	0.019	78 ± 8.2	78 ± 8.8	0.503	79.1 ± 8	76.1 ± 9	0.003	79.6 ± 7.8	76.7 ± 8.9	0.003
Male, *n* (%)		112 (50.2)	74 (64.9)	0.010	60 (43.5)	126 (63.3)	<0.001	75 (54.3)	111 (55.8)	0.795	110 (53.7)	76 (57.6)	0.480	88 (61.5)	98 (50.5)	0.044
BMI (kg/m^2^)		25.6 (22.7–28.9)	24.5 (24.5–27.6)	0.1	25.2 (22.7–28.7)	24.8 (22.8–28.4)	0.762	25.6 (22.7–28.7)	24.8 (22.8–28.3)	0.638	24.8 (22.5–28.7)	25.1 (23.3–28.1)	0.742	24.6 (22.3–27.5)	25.8 (25.8–29.4)	0.004
Euroscore II, %		5.1 (3.0–8.3)	4.7 (2.6–8.1)	0.299	5.5 (3.6–9.9)	4.3 (2.4–7.1)	<0.001	5.3 (3.1–9.5)	4.7 (2.7–7.6)	0.048	5.2 (3–9.3)	4.5 (2.6–7.9)	0.064	5.0 (2.8–8.1)	5.0 (2.9–8.5)	0.999
NYHA functional class, *n* (%)	I or II	10 (4.5)	24 (21.1)	<0.001	9 (6.5)	25 (12.6)	<0.001	6 (4.3)	28 (14.1)	<0.001	18 (8.8)	16 (12.1)	0.026	13 (9.1)	21 (10.8)	0.871
	III or IV	213 (95.5)	90 (79)		129 (93.5)	174 (87.4)		132 (95.7)	171 (85.9)		187 (91.2)	116 (87.8)		130 (90)	163 (89.2)	
Heart rate (bpm)		72 (65–82)	75 (66–82)	0.416	74 (65–83)	72 (65–81)	0.479	74 (65–87)	72 (65–80)	0.588	75 (65–83)	71 (65–82)	0.263	75 (67–84)	72 (65–81)	0.297
Systolic blood pressure (mmHg)		125 (110–139)	128 (115–140)	0.155	127 (114–138)	124 (110–140)	0.616	123 (110–136)	128 (110–140)	0.155	125 (113–140)	126 (110–140)	0.949	125 (109–140)	126 (114–140)	0.323
Comorbidities, *n* (%)
	Arterial hypertension	164 (73.5)	84 (73.7)	0.978	108 (78.3)	140 (70.4)	0.105	100 (72.5)	148 (74.4)	0.696	156 (76.1)	92 (69.7)	0.193	105 (73.4)	143 (73.7)	0.953
	Diabetes mellitus	53 (23.8)	30 (26.3)	0.607	39 (28.3)	44 (22.1)	0.198	38 (27.5)	45 (22.6)	0.302	51 (24.9)	32 (24.2)	0.895	41 (28.7)	42 (21.6)	0.139
	Previous stroke	30 (13.5)	20 (17.5)	0.318	21 (15.2)	29 (14.6)	0.870	20 (14.5)	30 (15.1)	0.882	30 (14.6)	20 (15.2)	0.896	21 (14.7)	29 (14.9)	0.946
	Previous myocardial infarction	59 (26.5)	33 (28.9)	0.627	37 (26.8)	55 (27.6)	0.867	42 (30.4)	50 (25.1)	0.282	55 (26.8)	37 (28)	0.809	39 (27.3)	53 (27.3)	0.992
	Coronary artery disease	136 (61.0)	63 (55.3)	0.312	83 (60.1)	116 (58.3)	0.734	87 (63)	112 (56.3)	0.214	121 (59)	78 (59.1)	0.990	86 (60.1)	113 (58.2)	0.727
	Carotid artery disease	11 (4.9)	5 (4.4)	1.0	8 (5.8)	8 (4.0)	0.604	8 (5.8)	8 (4.0)	0.604	9 (4.4)	7 (5.3)	0.794	3 (2.1)	13 (6.7)	0.068
	Previous cardiac surgery	72 (32.3)	46 (40.4)	0.142	38 (27.5)	80 (40.2)	0.017	43 (31.2)	75 (37.7)	0.217	63 (30.7)	55 (41.7)	0.040	47 (32.9)	71 (36.6)	0.478
Previous mitral valve surgery	11 (4.9)	4 (3.5)	0.549	4 (2.9)	11 (5.5)	0.250	5 (3.6)	10 (5.0)	0.539	6 (2.9)	9 (6.8)	0.091	4 (2.8)	11 (5.7)	0.206

	COPD	40 (17.9)	12 (10.5)	0.075	22 (15.9)	30 (15.1)	0.829	25 (18.1)	27 (13.6)	0.256	30 (14.6)	22 (16.7)	0.614	19 (13.3)	33 (17.0)	0.350
	Atrial fibrillation	151 (67.7)	61 (53.5)	0.011	99 (71.7)	113 (56.8)	0.005	89 (64.5)	123 (61.8)	0.616	134 (65.4)	78 (59.1)	0.244	90 (62.9)	122 (62.9)	0.992
	Tumour disease	36 (16.1)	20 (17.5)	0.744	20 (14.5)	36 (18.1)	0.383	23 (16.7)	33 (16.6)	0.984	34 (16.6)	22 (16.7)	0.984	23 (16.1)	33 (17.0)	0.821
	ICD	34 (15.2)	20 (17.5)	0.586	19 (13.8)	35 (17.6)	0.347	19 (13.8)	35 (17.6)	0.347	23 (11.2)	31 (23.5)	0.003	24 (16.8)	30 (15.5)	0.744
	CRT	40 (17.9)	18 (15.8)	0.621	20 (14.5)	38 (19.1)	0.271	26 (18.8)	32 (16.1)	0.509	35 (17.1)	23 (17.4)	0.934	33 (17.0)	25 (17.5)	0.910
	Dialysis	6 (2.7)	4 (3.5)	0.739	9 (6.5)	1 (0.5)	0.002	7 (5.1)	3 (1.5)	0.098	9 (4.4)	1 (0.8)	0.095	5 (3.5)	5 (2.6)	0.749
	History of cancer	53 (23.8)	27 (23.7)	1.0	31 (22.5)	49 (24.6)	0.697	35 (25.4)	45 (22.6)	0.604	50 (24.4)	30 (22.9)	0.794	38 (26.6)	42 (21.8)	0.365
Depression	10 (4.7)	2 (1.8)	0.351	6 (4.3)	6 (3.0)	0.560	7 (5.1)	5 (2.5)	0.243	9 (4.4)	3 (2.3)	0.380	7 (4.9)	5 (2.6)	0.373
Hb (g/dL)		12.1 ± 1.9	12.6 ± 1.7	0.02	11.7 ± 1.9	12.6 ± 1.7	<0.001	12.0 ± 1.9	12.4 ± 1.8	0.043	11.9 ± 1.8	12.6 ± 1.8	0.001	12.1 ± 1.7	12.3 ± 1.9	0.429
Platelets (×10^9^/L)		203 (164–245)	197 (153–230)	0.174	209 (164–247)	193 (155–236)	0.073	210 (166–249)	190 (153–232)	0.037	204 (163–243)	193 (157–239)	0.649	190 (151–231)	205 (165–243)	0.102
LDL (mg/dL)		85 (60–106)	91 (67–124)	0.200	92 (62–114)	85 (63–107)	0.606	89 (61–107)	88 (63–112)	0.961	89 (60–107)	85 (65–117)	0.637	80 (60–103)	93 (65–114)	0.097
HDL (mg/dL)		47 (38–61)	46 (38–60)	0.969	47 (36–60)	47 (39–61)	0.422	47 (36–58)	47 (39–63)	0.247	47 (37–60)	47 (38–61)	0.612	47 (36–57)	47 (38–63)	0.348
Non‐HDL cholesterol (mg/dL)		103 (74–126)	108 (86–143)	0.07	107 (78–128)	105 (76–133)	0.635	104 (80–126)	105 (75–133)	0.857	107 (78–126)	103 (76–135)	0.708	97 (76–119)	112 (78–137)	0.085
TSH (mIU/L)		1.7 (1.0–2.7)	1.3 (1.3–2.2)	0.011	1.7 (1.0–2.6)	1.5 (0.9–2.5)	0.303	1.7 (1.0–2.9)	1.5 (0.9–2.4)	0.139	1.7 (0.9–2.6)	1.5 (0.9–2.6)	0.660	1.6 (1.0–2.5)	1.5 (0.92–2.6)	0.981
eGFR (mL/min/m^2^)		44 (31–60)	43 (34–62)	0.478	38 (25–49)	49 (37–65)	<0.001	40 (26–59)	44 (36–61)	0.015	41 (29–59)	46 (37–61)	0.008	44 (33–61)	44 (32–60)	0.751
NT‐proBNP (ng/L)		5327 ± 8142	4777 ± 8427	0.008	7088 ± 11 148	3781 ± 4930	<0.001	6516 ± 11 029	4204 ± 5427	0.002	5989 ± 9781	3837 ± 4753	0.041	5403 ± 9248	4954 ± 7462	0.159
Cause of MR, *n* (%)	Degenerative	83 (37.2)	44 (38.9)	0.584	58 (42)	69 (34.8)	0.393	45 (32.6)	82 (41.4)	0.260	84 (41)	43 (32.8)	0.316	51 (35.7)	76 (39.4)	0.383
	Functional	121 (54.3)	56 (49.6)		67 (48.6)	110 (55.6)		79 (57.2)	98 (49.5)		103 (50.2)	74 (56.5)		81 (56.6)	96 (49.7)	
	Combined pathology	19 (8.5)	13 (11.5)		13 (9.4)	19 (9.6)		14 (10.1)	18 (9.1)		18 (8.8)	14 (10.7)		11 (7.7)	21 (10.9)	
Echocardiography grade of MR, *n* (%)	≤III	51 (22.9)	22 (19.3)	0.451	23 (16.7)	50 (25.1)	0.064	31 (22.5)	42 (21.1)	0.766	39 (19.0)	34 (25.8)	0.143	29 (20.3)	44 (22.7)	0.597
	IV	172 (77.1)	92 (80.7)		115 (83.3)	149 (74.9)		107 (77.5)	157 (78.9)		166 (81.0)	98 (74.2)		114 (79.7)	150 (77.3)	
Echocardiography LVEF, *n* (%)	>50%	109 (48.9)	57 (50)	0.975	73 (52.9)	93 (46.7)	0.269	62 (44.9)	104 (52.3)	0.223	105 (51.2)	61 (46.2)	0.132	70 (49.0)	96 (49.5)	0.962
	30–50%	65 (29.1)	33 (28.9)		41 (29.7)	57 (28.6)		40 (29.0)	58 (29.1)		63 (30.7)	35 (26.5)		41 (28.7)	57 (29.4)	
	<30%	49 (22.0)	24 (21.1)		24 (17.4)	49 (24.6)		36 (26.1)	37 (18.6)		37 (18.0)	36 (27.3)		32 (22.4)	41 (21.1)	
Echocardiography	LAVI (ml/m^2^)	56 (43–72)	62 (50–83)	0.005	61 (47–78)	56 (44–73)	0.156	57 (44–72)	59 (44–77)	0.452	59 (45–77)	57 (44–71)	0.331	58 (43–74)	58 (45–76)	0.54
	LVEDD (mm)	54 (48–61)	55 (50–62)	0.251	54 (49–59)	55 (44–73)	0.037	54 (48–60)	55 (48–62)	0.353	54 (48–60)	55 (49–63)	0.083	55 (49–61)	54 (48–62)	0.523
	MPG (mmHg)	2.4 ± 1.0	2.8 ± 1.4	0.096	2.7 ± 1.0	2.5 ± 1.3	0.022	2.4 ± 1.0	2.7 ± 1.3	0.176	2.7 ± 1.3	2.4 ± 1.0	0.078	2.6 ± 1.2	2.5 ± 1.2	0.883
	sPAP (mmHg)	55 (45–68)	54 (45–65)	0.429	53 (45–64)	56 (45–66)	0.560	53 (45–64)	55 (45–66)	0.707	55 (45–68)	52 (44–64)	0.316	53 (45–65)	56 (45–66)	0.255

*Note:* Entries printed in bold are for improved readability. Abbreviations: BMI = body mass index; bpm = beats per minute; CRT = cardiac resynchronization therapy; COPD = chronic obstructive pulmonary disease; Hb = haemoglobin; ICD = implantable cardioverter defibrillator; eGFR = estimated glomerular filtration rate; HDL = high‐density lipoprotein; LDL = low‐density lipoprotein; NT‐proBNP = N‐terminal pro‐brain natriuretic peptide; MR = mitral regurgitation; LAVI = left atrial volume index; LVEDD = left ventricular end‐diastolic diameter; LVEF = left ventricular ejection fraction; MPG = mean pressure gradient; NYHA = New York Heart Association; sPAP = systolic pulmonary artery pressure; TSH = thyroid‐stimulating hormone.

Since distinct frailty domains showed no association with mortality or no independent association in analysis including all five domains, a new and simplified definition of frailty (S‐frailty) was proposed using only domains with an independent association with mortality. Primary analysis used for derivation of S‐frailty was executed in patients enrolled till October 2017, and patients enrolled from November 2017 to December 2019 were used for validation of S‐frailty.

To assess the additive prognostic impact of S‐frailty in comparison to the MitraScore, we generated a logistic regression model with 1‐year mortality as the primary outcome as well as a Cox regression model with mortality as the primary outcome and both with MitraScore as a univariate predictor, and subsequently with S‐frailty in a bivariate model. The respective predicted probabilities or hazard ratios (HRs) were used to calculate the receiver operating characteristic area under the curve (AUC) or Harrell's c‐statistic, respectively. Additionally, net reclassification index (for risk categories <10%, 10%–20% and >20%) and integrated discrimination improvement were calculated for 1‐year mortality. These calculations were carried out in Stata 17.0. All other statistical analyses were carried out in SPSS Software (Version 28.0). Statistically significant *p* values were defined as < 0.05.

## 3 Results

Six hundred two patients with written informed consent were eligible within the study period. Two patients died of terminal heart failure before the intervention. Seventy‐six patients were excluded because of incomplete data on frailty domains. Thus, 524 patients were included in the study. Mean age (standard deviation) was 77 (9) years, and 54.6% were male. Median Euroscore II was 5.3%. Four hundred fifty‐eight patients (87.4%) underwent edge‐to‐edge mitral valve repair with MitraClip and 32 patients (6.1%) with PASCAL, with combined mitral and tricuspid valve repair in 23 cases (4.4%). Direct annuloplasty with Cardioband was performed in 11 patients (2.1%). Three hundred thirty‐seven patients were selected for analyzing the prognostic impact of individual frailty domains and derivation of a new frailty measure based on outcome prediction and this measure was validated in an additional 187 patients. There were no relevant differences between the derivation and the validation cohort despite a slightly higher Euroscore II in the validation cohort (6.6 vs. 5.0) (Supp. Table S2).

### 3.1 Frailty Domains

In the derivation cohort 164 of 337 patients (48.7%) were classified as frail according to Fried. Two hundred twenty‐three patients (66.2%) showed exhaustion, 138 patients (40.9%) each showed slowness and inactivity, 205 patients (60.8%) showed weakness, and 143 patients (42.4%) showed unintended weight loss. There were no significant differences in the aetiology of MR and comorbidities between patients with and without the respective five frailty domains (Table 1). Exhausted, slow, inactive and weak patients presented with significantly more severe heart failure assessed by NYHA class and NT‐proBNP levels than patients without the respective frailty domains. Slow and inactive patients had a higher median Euroscore II compared to patients without the respective domains. Patients with weight loss were significantly older but otherwise did not differ from patients without weight loss.

322 (95.5%) procedures were technically successful, with 289 (85.8%) showing a reduction of MR to grade 2 or lower at discharge. No intraprocedural death or emergency cardiac surgery occurred. Eight patients (2.4%) died within the first 6 weeks after the procedure, and six patients (1.8%) had a stroke during the procedural hospital stay. There were no differences in procedural complications, reduction of MR at discharge and 6 weeks mortality between patients with and without the respective frailty domains (Supp. Table S1).

**FIGURE 1:**
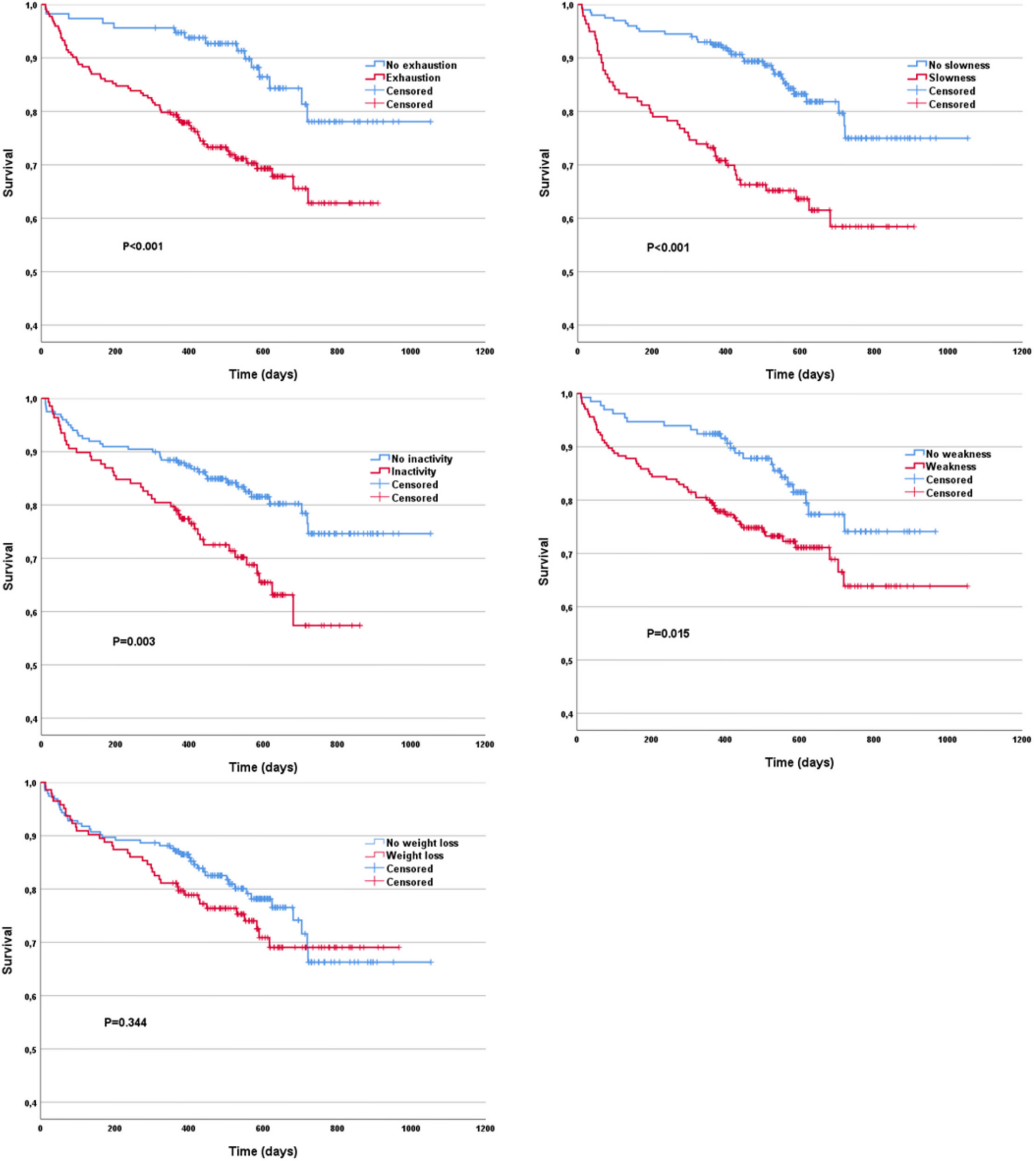
Kaplan–Meier survival plots by individual frailty domains. *p* value by log‐rank test.

At a median follow‐up of 517 days (IQR: 379–621 days), 80 patients (23.7%) died, and 64 patients (19%) were hospitalized for heart failure. A total of 249 patients (73.9%) survived free of heart failure hospitalization (HFH) at 1 year. Exhausted, slow, inactive, and weak patients showed significantly increased 1‐year mortality with absolute differences of 9.4%–19.3% compared to patients without the respective domain (Table 2). One‐year mortality did not differ by weight loss. The HRs of death ranged from 1.83 to 2.74 for exhausted, slow, inactive and weak patients (Figure 1, Table 2). The HR of mortality or HFH ranged from 1.61 to 1.92 for slow, inactive and exhausted patients. Mortality or HFH did not differ by weakness or weight loss. In multivariate Cox regression analysis of mortality and mortality or HFH including all five individual frailty domains only exhaustion and slowness remained significant.

**TABLE 2:** Clinical outcome by individual frailty domains.

	Exhaustion			Slowness			Inactivity			Weakness			Weight loss		
	Positive *n* = 223	Negative *n* = 114	*p*	Positive *n* = 138	Negative *n* = 199	*p*	Positive *n* = 138	Negative *n* = 199	*p*	Positive *n* = 205	Negative *n* = 132	*p*	Positive *n* = 143	Negative *n* = 194	*p*
Death < 1 year, *n* (%)	46 (20.6)	6 (5.3)	<0.001	37 (26.8)	15 (7.5)	<0.001	29 (21)	23 (11.6)	0.018	42 (20.5)	10 (7.6)	0.001	27 (18.9)	25 (12.9)	0.132
Death, unadjusted HR (95% CI)	2.24 (1.63–3.08)		<0.001	2.74 (1.75–4.30)		<0.001	1.96 (1.26–3.05)		0.003	1.83 (1.12–2.96)		0.015	1.24 (0.80–1.92)		0.344
Death, adjusted* HR (95% CI)	1.97 (1.10–3.60)		0.028	1.98 (1.21–3.23)		0.007	1.26 (0.78–2.03)		0.35	1.45 (0.87–2.39)		0.151	1.2 (0.77–1.86)		0.43
Death or heart failure hospitalization < 1 year, *n* (%)	70 (31.4)	18 (15.8)	0.002	51 (37.0)	37 (18.6)	<0.001	44 (31.9)	44 (22.1)	0.045	64 (31.2)	24 (18.2)	0.008	41 (28.7)	47 (24.2)	0.359
Death or heart failure hospitalization, unadjusted HR (95% CI)	1.83 (1.21–2.77)		0.004	1.92 (1.34–2.75)		<0.001	1.61 (1.12–2.31)		0.011	1.34 (0.92–1.95)		0.129	1.12 (0.78–1.61)		0.542
Death or heart failure hospitalization, adjusted* HR (95% CI)	1.49 (0.95–2.33)		0.084	1.60 (1.07–2.38)		0.021	1.22 (0.83–1.81)		0.317	1.11 (0.74–1.64)		0.622	1.10 (0.76–1.57)		0.637

* Adjusted for Euroscore II, continuous ln NT‐proBNP (N‐terminal pro‐brain natriuretic peptide), eGFR (estimated glomerular filtration rate), arterial hypertension, coronary artery disease, LVEDD (left ventricular end‐diastolic diameter).

### 3.2 Simplified Frailty Measure

**FIGURE 2:**
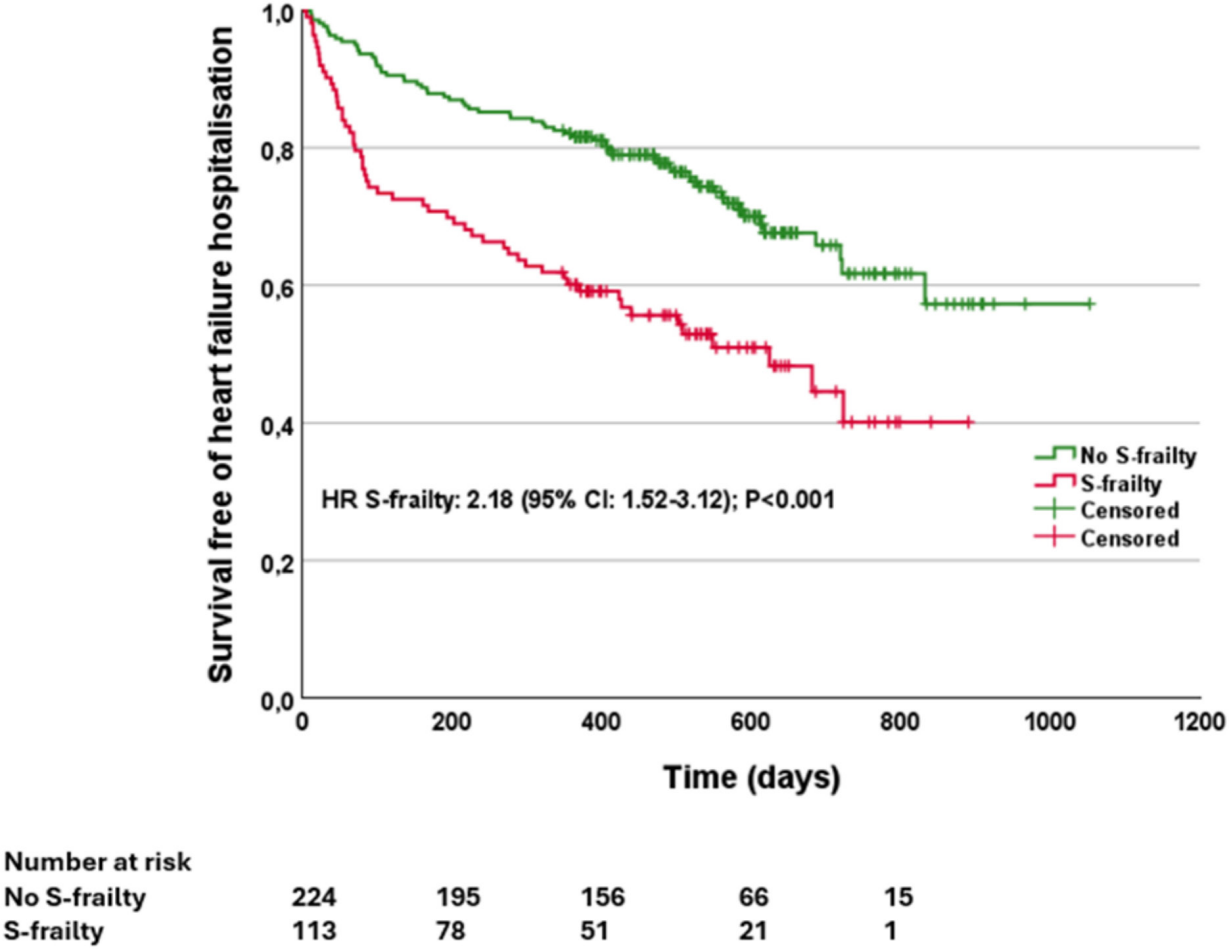
Kaplan–Meier survival free of heart failure hospitalization plot by simplified‐frailty (S‐frailty). *p* value by log‐rank test.

S‐frailty was calculated from the domains exhaustion and slowness and defined if both domains were present concomitantly (in *n* = 113 [33.5%] patients). Patients with none or only one of the two domains were defined as not S‐frail (*n* = 224 [66.5%] patients). S‐frail patients had a significantly increased risk of mortality and mortality or HFH (Central Illustration, Figure 2, Table 3), with a 1‐year event rate of 30.1% for death and 39.8% for death or HFH in S‐frail patients compared to 8.03% and 18.3%, respectively in non–S‐frail patients. After adjustment for other clinical risk factors S‐frailty remained significantly associated with adverse outcome, with a HR of 4.45 (95% CI: 2.42–8.19) for mortality and a HR of 2.60 (95% CI: 1.64–4.11) for mortality or HFH (Table 3). There were also no changes in the results after adjustment for residual MR after the intervention. S‐frailty showed similar risk discrimination in the derivation and validation cohort (Central Illustration, Supp. Table S3, Supp. Figure S1).

**TABLE 3:** Univariate and multivariate Cox regression analysis of baseline characteristics and simplified frailty.

	Univariate analysis	Death, HR (95% CI)	*p*	Death or heart failure rehospitalization, HR (95% CI)	*p*	Multivariate analysis	Death, HR (95% CI)	*p*	Death or heart failure rehospitalization, HR (95% CI)	*p*
Euroscore II		1.06 (1.02–1.09)	<0.001	1.03 (1.01–1.06)	0.021		1 (0.97–1.04)	0.836	0.99 (0.96–1.03)	0.602
Continuous ln NT‐proBNP		5.08 (3.07–8.4)	<0.001	2.93 (2.00–4.40)	<0.001		1.97 (1.47–2.64)	<0.001	1.46 (1.16–1.82)	0.001
eGFR		0.97 (0.96–0.98)	<0.001	0.98 (0.97–0.99)	<0.001		1 (0.99–1.02)	0.531	1.00 (0.99–1.02)	0.792
Arterial hypertension		0.57 (0.36–0.89)	0.014	0.72 (0.49–1.05)	0.087		0.46 (0.26–0.81)	0.007	0.61 (0.39–0.96)	0.032
Coronary artery disease		1.67 (1.05–2.73)	0.032	1.26 (0.87–1.83)	0.229		2.19 (1.16–4.15)	0.016	1.47 (0.92–2.35)	0.109
LVEDD		1.03 (1.00–1.05)	0.042	1.02 (1.00–1.04)	0.021		1.03 (1–1.07)	0.037	1.03 (1.00–1.05)	0.028
Simplified frailty		3.21 (2.06–5)	<0.001	2.17 (1.52–3.12)	<0.001		4.45 (2.42–8.19)	<0.001	2.60 (1.64–4.13)	<0.001

Abbreviations: eGFR = estimated glomerular filtration rate; LVEDD = left ventricular end‐diastolic diameter; NT‐proBNP = N‐terminal pro‐brain natriuretic peptide.

### 3.3 Predictive Value of S‐Frailty Beyond the MitraScore

In the total study cohort combining derivation and validation groups, the MitraScore [6] was significantly associated with mortality with a HR of 1.49 (95% CI: 1.30–1.70; *p* < 0.001) and with mortality or HFH with an HR of 1.43 (95% CI: 1.25–1.64; *p* < 0.001) per score point. When combining the MitraScore and S‐frailty as predictors in a Cox regression model, S‐frailty remained significantly associated with mortality (HR: 2.57; 95% CI: 1.78–3.71, *p* < 0.001) and the combined endpoint of mortality or HFH (HR: 1.90; 95% CI: 1.32–2.74; *p* < 0.001). Prediction of mortality risk was improved in a model with both S‐frailty and MitraScore compared to MitraScore alone, improving Harrell's C statistic by 0.054 (Table 4). Net reclassification index (0.21, standard error 0.08, *p* = 0.012) and integrated discrimination index (0.07, standard error 0.01, *p* < 0.001) demonstrated a significant improvement in the prediction of 1‐year mortality when adding S‐frailty to the MitraScore.

**TABLE 4:** Simplified‐frailty as predictor in combination with MitraScore.

	AUC (logistic regression with 1‐year mortality)	Confidence interval	*p* versus respective score without S‐frailty	Harrell's c‐statistic (Cox regression)	Confidence interval	*p* versus respective score without S‐frailty
MitraScore	0.6775	0.6237–0.7313	—	0.6454	0.5940–0.6967	—
MitraScore + S‐frailty	0.7160	0.6628–0.7692	0.047	0.7000	0.6493–0.7507	0.002

Patients with high MitraScore (supramedian, > 3 points) had significantly increased risk of mortality (HR: 2.82, 95% CI: 1.95–4.07, *p* < 0.001). When patients were further stratified by S‐frailty, patients with high MitraScore and S‐frailty had the highest risk of mortality compared to patients with low MitraScore and no S‐frailty, and patients with high MitraScore and no S‐frailty (HR: 2.83, 95% CI: 1.75–4.59) while patients with low MitraScore and S‐frailty had intermediate risk (HR: 2.48, 95% CI: 1.40–4.38) (Central Illustration).

## 4 Discussion

This study is the first to examine the impact of individual frailty domains on outcomes in patients undergoing PMVR. The association with long‐term clinical outcomes substantially differed across frailty domains, with a significant association with mortality and mortality or HFH observed for exhaustion, slowness, inactivity and weakness but not weight loss. Within the domains, only exhaustion and slowness showed an independent association with adverse outcomes, and we propose an S‐frailty measure based on these two domains. S‐frailty was associated with mortality and mortality or HFH after adjustment for other clinical risk factors and significantly improved risk prediction of the MitraScore.

Most scores evaluated for risk stratification in patients undergoing PMVR so far showed weak or moderate performance [3, 4, 5, 19, 20], which is not unexpected given that the majority of these scores were not derived from PMVR cohorts. The MitraScore is one of very few scores that was originally derived from a large population undergoing PMVR and validated in an independent cohort [6]. Even though the discriminatory performance of the MitraScore is moderate. A likely explanation is that only clinical and demographic variables were considered that were retrospectively available from routine assessments. However, patients selected for PMVR show a broad spectrum and combination of cardiovascular and noncardiovascular diseases of different severity potentially accompanied by aging‐related processes. Such complex interplay of pathologies is difficult to cover by a pragmatic clinical score comprising a small number of variables.

Contrarily, the concept of frailty can be regarded as a global summary phenotype resulting from deficits in different physiological systems finally contributing to a failure of homeostatic mechanisms [21]. A huge body of evidence supports the strong and additive prognostic role of the frailty syndrome not only in elderly populations but also in cohorts with a variety of relevant organ morbidity [22, 23, 24]. For instance, frailty substantially improved risk discrimination of the MAGGIC score in a heart failure population [25]. Similarly, in patients undergoing transcatheter aortic valve replacement (TAVR), frailty improved risk prediction of the Euroscore and STS score [26].

The frailty model by Fried was developed from the prognostic impact of distinct functional domains in an elderly, population‐based cohort [9]. Frailty was finally defined by summing up the five equally weighted domains. Albeit this frailty measure proved to be of high prognostic relevance in many cardiovascular populations such as acute and chronic heart failure, TAVR and PMVR, the impact of distinct domains might differ across populations and outcomes of interest. For instance, a study on patients hospitalized for heart failure showed that only four of the five Fried domains were predictive of mortality and the weight of individual domains differed by a factor of 2.5 [27].

In patients with a background of heart failure, validation of individual frailty domains is highly expedient given that there is a large phenotypical overlap between physical frailty and heart failure. For example, unintentional weight loss was not associated with mortality and heart failure hospitalization in our and other cohorts with heart failure [28]. Albeit patients with heart failure measure body weight regularly, unintended weight loss has been shown to be a strong predictor of poor prognosis in chronic heart failure [29]. The self‐assessment of long‐term changes of body weight and in consequence unintended weight loss might be inaccurate considering the strong dynamics of body weight by shifts in volume status [30].

Another point to consider when assessing patients before PMVR is that valve repair can modify the expression of certain domains and hence their prognostic value. We have shown that PMVR significantly improves the domains slowness, exhaustion and inactivity but not weakness [15]. One might expect that weakness, since unaffected by PMVR, is a particularly robust risk marker. However, the association of exhaustion and slowness with adverse outcomes was stronger than that of weakness in our cohort. Basically, both weakness and slowness are determined by sarcopenia and loss of muscle mass. Nonetheless, several studies demonstrated that measures of lower body weakness such as gait speed are more strongly associated with overall frailty, functional decline and prognosis than weakness assessed by hand dynamometer [31, 32, 33, 34, 35]. One likely explanation is that slowness concomitantly represents dysfunction in multiple organ systems, such as cardiovascular, pulmonary, nervous and musculoskeletal [36].

Differences in the prognostic weight of individual frailty domains observed across studies might also be attributable to the tools of assessment. We used a simplified assessment of inactivity when compared to the initial report by Fried where an extensive questionnaire on physical activity was used. However, tools aimed to be applied in clinical routine need to be rapid and easy, and the accuracy of assessment needs to be balanced against feasibility.

The relevance of frailty in the management of patients with cardiovascular disease is undisputable [37, 38, 39] and the prognostic impact of frailty for different cardiovascular outcomes has been extensively demonstrated [40]. However, particularly in the context of limited resources in routine care assessment of frailty needs to be adapted to patient characteristics and clinical aims. Our study provides important first evidence supporting routine frailty assessment for risk stratification in PMVR as a basis for shared decision making with patients. We demonstrated that for this situation only two of the five frailty domains provide independent information regarding mortality and HFH, which will enable simple and rapid assessment by asking one question and measuring time of walking through a room. Importantly, this frailty measure added prognostic information to clinical parameters and improved risk discrimination of the MitraScore, which was specifically developed for PMVR patients.

## 5 Limitations

This was a monocentre study, which might limit the generalizability of results. However, our study population was comparable to other PMVR registries in terms of comorbidities, age, Euroscore, procedural results and outcomes [41, 42]. Furthermore, the large sample size allowed independent validation of the risk discrimination performance of the S‐frailty definition. Although the Fried scale is one of the most used frailty assessment tools and is well validated, it is restricted to physical impairment and other frailty domains such as cognitive impairment or social deprivation need further evaluation. We have recently shown that a multidimensional prognostic index based on a comprehensive geriatric assessment is additive to the Fried phenotype in predicting risk in PMVR patients [13]. However, from the clinical perspective, the additive gain of information must be wisely balanced with limited resources for the assessment process. A comprehensive geriatric assessment is unlikely to be available for every patient undergoing PMVR.

## 6 Future Directions

Our findings highlight the need for validation in independent, preferably multicenter cohorts, and for prospective interventional studies to evaluate whether incorporating this simplified frailty measure into clinical algorithms can improve procedural outcomes, optimize patient management, and guide healthcare resource allocation. These findings may extend beyond PMVR, guiding future research on the integration of functional and geriatric assessments into cardiovascular care and other high‐risk interventions.

## 7 Conclusions

Individual frailty domains are of variable prognostic relevance in patients undergoing PMVR. An independent association with long‐term mortality and HFH was observed only for exhaustion and slowness. An S‐frailty definition based on these domains showed a strong association with adverse outcomes when adjusted for other risk factors and significantly improved risk prediction of the well‐established MitraScore. Our findings provide the first evidence supporting the integration of frailty measures in the risk estimation of patients undergoing PMVR.

## Funding

The authors received no specific funding for this work.

## Conflicts of Interest

The authors declare no conflicts of interest.

## Supporting information


**Table S1:** Procedural results by status of individual frailty domains.
**Table S2:** Baseline characteristics of derivation and validation group.
**Table S3:** Sensitivity, specificity, and predictive value of derivation and validation group.
**Figure S1:** Kaplan–Meier survival plot by simplified‐frailty (S‐frailty) (Validation cohort) *p* value by log‐rank test.
**Figure S2:** Study flowchart.

**Data S1:** Supplementary information.
