# Supplementary material for: A Simplified Frailty Measure Improves Risk Stratification in Patients Undergoing Percutaneous Mitral Valve Repair
Source: J Cachexia Sarcopenia Muscle. 2025 Dec 1;16(6):e70138. doi: 10.1002/jcsm.70138 (PMC12668899; doi:10.1002/jcsm.70138)
Supplement: Supplementary file 1 — Table S1: Procedural results by status of individual frailty domains. Table S2: Baseline characteristics of derivation and validation group. Table S3: Sensitivity, specificity, and predictive value of derivation and validation group. Figure S1: Kaplan–Meier survival plot by simplified‐frailty (S‐frailty) (Validation cohort) p value by log‐rank test. Figure S2: Study flowchart. [file JCSM-16-e70138-s002.docx]

**Supplemental files are intended for publication as an online data supplement.**

Supplemental Table 1 Procedural results by status of individual frailty domains

|  | | | Exhaustion | | | Slowness | | | Inactivity | | | Weakness | | | Weight loss | | |
| --- | --- | --- | --- | --- | --- | --- | --- | --- | --- | --- | --- | --- | --- | --- | --- | --- | --- |
|  | | | positive  n=223 | negative  n=114 | p value | positive  n=138 | negative  n=199 | p value | positive  n=138 | negative  n=199 | p value | positive  n=205 | negative  n=132 | p value | positive  n=143 | negative  n=194 | p value |
| Procedural death, no. | | | 0 | 0 |  | 0 | 0 |  | 0 | 0 |  | 0 | 0 |  | 0 | 0 |  |
| Death at 6 weeks, no. (%) | | | 6 (2.7) | 2 (1.8) | 0.722 | 6 (4.3) | 2 (1.0) | 0.068 | 3 (2.2) | 5 (2.5) | 1.0 | 7 (3.4) | 1 (0.8) | 0.155 | 3 (2.1) | 5 (2.6) | 1.0 |
| Technical success, no. (%) | | | 214 (96.0) | 108 (94.7) | 0.605 | 133 (96.4) | 189 (95.0) | 0.539 | 136 (98.6) | 186 (93.5) | 0.026 | 195 (95.1) | 127 (96.2) | 0.636 | 139 (97.2) | 183 (94.3) | 0.206 |
| Device not implanted, no. (%) | | | 9 (4.0) | 6 (5.3) |  | 5 (3.6) | 10 (5.0) |  | 2 (1.4) | 13 (6.5) |  | 10 (4.9) | 5 (3.8) |  | 4 (2.8) | 11 (5.7) |  |
| Procedural complications | | |  |  |  |  |  |  |  |  |  |  |  |  |  |  |  |
| Stroke, no. (%) | Stroke, no. (%) | | 4 (1.8) | 2 (1.8) | 1.000 | 3 (2.2) | 3 (1.5) | 0.692 | 2 (1.4) | 4 (2.0) | 1.000 | 4 (2.0) | 2 (1.5) | 1.000 | 5 (3.5) | 1 (0.5) | 0.087 |
|  | Pericardial effusion necessitating pericardiocentesis,  no. (%) | | 1 (0.4) | 1 (0.9) | 1.000 | 1 (0.7) | 1 (0.5) | 1.000 | 0 | 2 (1.0) | 0.515 | 1 (0.5) | 1 (0.8) | 1.000 | 2 (1.4) | 0 | 0.099 |
|  | Vascular complications with intervention, no. (%) | | 6 (2.7) | 2 (1.8) | 0.722 | 5 (3.6) | 3 (1.5) | 0.280 | 3 (2.2) | 5 (2.5) | 1.000 | 7 (3.4) | 1 (0.8) | 0.155 | 2 (1.4) | 6 (3.1) | 0.475 |
| Length of hospital stay, days | | | 8 (6-10) | 7 (6-10) | 0.059 | 9 (7-11) | 7 (6-10) | 0.001 | 8 (6-11) | 8 (6-10) | 0.024 | 8 (6-11) | 7 (5-9) | <0.001 | 8 (6-10) | 8 (6-10) | 0.610 |
| Discharge echocardiography | | |  |  |  |  |  |  |  |  |  |  |  |  |  |  |  |
|  | LV-EF (%) | | 45 ± 15 | 45 ± 16 | 0.779 | 46 ± 15 | 44 ± 16 | 0.253 | 43 ± 16 | 46 ± 15 | 0.042 | 45 ± 15 | 44 ± 15 | 0.376 | 45 ± 15 | 45 ± 15 | 0.956 |
|  | MR | |  |  | 0.097 |  |  | 0.960 |  |  | 0.201 |  |  | 0.199 |  |  | 0.087 |
|  |  | Minimal,  no. (%) | 9 (4.1) | 8 (7.1) |  | 6 (4.5) | 11 (5.6) |  | 8 (5.9) | 9 (4.6) |  | 9 (4.5) | 8 (6.2) |  | 12 (8.5) | 5 (2.6) |  |
|  |  | I°, no. (%) | 120 (54.8) | 49 (43.8) |  | 70 (52.2) | 99 (50.3) |  | 76 (56.3) | 93 (47.4) |  | 108 (53.5) | 61 (47.3) |  | 67 (47.2) | 102 (54) |  |
|  |  | II°, no. (%) | 72 (32.9) | 49 (43.8) |  | 48 (35.8) | 73 (37.1) |  | 45 (33.3) | 76 (38.8) |  | 67 (33.2) | 54 (41.9) |  | 51 (35.9) | 70 (37) |  |
|  |  | III-IV°, no. (%) | 18 (8.2) | 6 (5.4) |  | 10 (7.5) | 14 (7.1) |  | 6 (4.4) | 18 (9.2) |  | 18 (8.9) | 6 (4.7) |  | 12 (8.5) | 12 (6.3) |  |
| Discharge to a rehabilitation institution, no. (%) | | | 115 (51.6) | 53 (46.5) | 0.313 | 71 (51.4) | 97 (48.7) | 0.433 | 69 (50.0) | 99 (49.7) | 0.995 | 100 (48.7) | 68 (51.5) | 0.734 | 72 (50.3) | 96 (49.5) | 0.923 |

LVEF=Left ventricular ejection fraction; MR=Mitral regurgitation

Supplemental Table 2 Baseline characteristics of derivation and validation group

|  |  | Derivation cohort  (n=337) | Validation cohort  (n=187) | p value |
| --- | --- | --- | --- | --- |
| Age, years |  | 77.9 ± 8.6 | 76.2 ± 9.9 | 0.089 |
| Male, no. (%) |  | 186  (55.2) | 100  (53.5) | 0.715 |
| Bodymass Index, kg/m² |  | 30.0 ± 4.9 | 25.0 ± 4.6 | 0.068 |
| Euroscore II, % |  | 5.0  (2.8 – 8.3) | 6.6  (3.7 – 14.7) | <0.001 |
| NYHA functional class,  no. (%) | I or II | 34  (10.1) | 23  (12.3) | 0.387 |
|  | III or IV | 303  (89.9) | 160  (85.6) |  |
| Comorbidities, no. (%) | Arterial hypertension | 248  (73.6) | 144  (77) | 0.238 |
|  | Diabetes mellitus | 83  (24.6) | 47  (25.1) | 0.871 |
|  | Previous myocardial infarction | 92  (27.3) | 41  (21.9) | 0.262 |
|  | Coronary artery disease | 199  (59.1) | 101  (54) | 0.293 |
|  | Previous cardiac surgery | 118  (35) | 64  (34.2) | 0.797 |
|  | COPD | 52  (15.4) | 21  (11.2) | 0.191 |
|  | Atrial fibrillation | 212  (62.9) | 134  (71.7) | 0.043 |
|  | Tumor disease | 56  (16.6) | 43  (23) | 0.074 |
|  | ICD | 54  (16) | 34  (18.2) | 0.441 |
|  | CRT | 58  (17.2) | 45  (24.1) | 0.041 |
| eGFR (ml/min/m²) |  | 44  (32 – 60) | 45  (34 – 62) | 0.207 |
| NT-proBNP (ng/l) |  | 2523  (1468 – 5681) | 2228  (1157 – 5801) | 0.253 |
| Cause of MR, no. (%) | Degenerative | 127  (37.7) | 57  (39.9) | 0.594 |
|  | Functional | 177  (52.5) | 69  (36.9) |  |
|  | Combined pathology | 32  (9.5) | 17  (9.1) |  |
| Echocardiography  LVEF, no. (%) | >50% | 166  (49.3) | 57  (30.5) | 0.413 |
|  | 30-50% | 98  (29.1) | 45  (24.1) |  |
|  | <30% | 73  (21.7) | 32  (17.1) |  |
| MLwHFQ Score |  | 31  (22 – 43) | 38  (21 – 56) | 0.016 |
| MitraScore |  | 3  (2-4) | 3  (2-4) | 0.211 |

NYHA=New York Heart Association; COPD=Chronic obstructive pulmonary disease; ICD=Implantable cardioverter defibrillator; CRT=Cardiac resynchronization therapy; eGFR=Estimated glomerular filtration rate; NT-proBNP=N-terminal pro-brain natriuretic peptide; MR=Mitral regurgitation; LVEF=Left ventricular ejection fraction; MLwHFQ=Minnesota living with heart failure questionnaire

Supplemental Table 3 Sensitivity, specificity, and predictive value of derivation and validation group

|  |  | Derivation cohort (n=337) | Validation cohort (n=187) |
| --- | --- | --- | --- |
| Death <1 year |  |  |  |
|  | Sensitivity (%) | 65.4 | 48.4 |
|  | Specificity (%) | 72.3 | 81.3 |
|  | Positive predictive value (%) | 30.1 | 38.5 |
|  | Negative predictive value (%) | 92.0 | 86.7 |
| Death or heart failure hospitalization <1 year |  |  |  |
|  | Sensitivity (%) | 52.3 | 43.6 |
|  | Specificity (%) | 73.1 | 90.4 |
|  | Positive predictive value (%) | 40.7 | 77.3 |
|  | Negative predictive value (%) | 81.3 | 68.1 |

*Supplemental Figure 1 Kaplan-Meier survival plot by simplified-frailty (S-frailty) (Validation cohort)*

*p value by log-rank test*


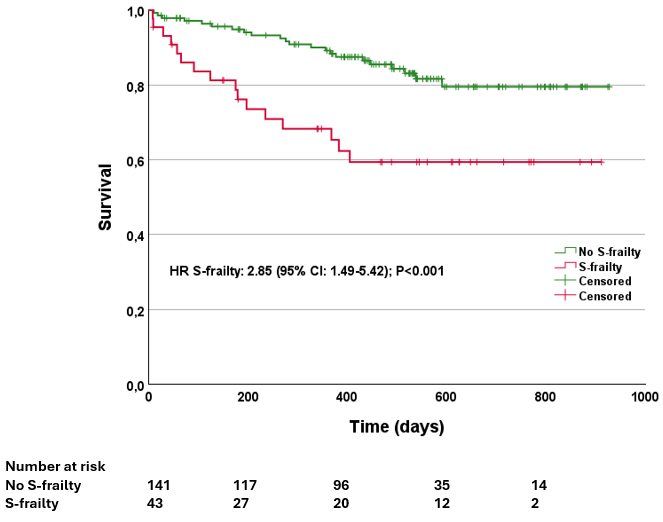


*Supplemental Figure 2 Study Flowchart*

PMVR procedure started (n=524)

Discharged alive (n=519)

In-hospital death postprocedural (n=5)

Patients admitted for PMVR (n=602)

Missing Frailty criteria (n=76)

Preprocedural death (n=2)

Lost to follow-up (n=0)

Long time Follow up data (Medium 517 d)
